# [Corrigendum] Cerebral ischemic post-conditioning induces autophagy inhibition and a HMGB1 secretion attenuation feedback loop to protect against ischemia reperfusion injury in an oxygen glucose deprivation cellular model

**DOI:** 10.3892/mmr.2025.13641

**Published:** 2025-07-30

**Authors:** Jue Wang, Dong Han, Miao Sun, Juan Feng

Mol Med Rep 14: 4162–4172, 2016; DOI: 10.3892/mmr.2016.5747

Subsequently to the publication of the above paper, the authors drew to the Editor's attention that matching HMGB1 bands featured in Figs. 4 and 5 had inadvertently been included in these figures twice; moreover, the GAPDH western blot controls featured in Fig. 3 had also been selected incorrectly.

Revised versions of Fig. 3, 4 and 5, now featuring the corrected GAPDH western blots in Fig. 3, replacement data for the Beclin1 blots and the GAPDH blots in Fig. 4B and the HGMB-1 bands in Fig. 4F, and replacement blots for the free beclin-1 and HMGB-1 bands in Fig. 5, are shown on the next three pages. Note that these errors did not adversely affect either the results or the overall conclusions reported in this study. All the authors agree with the publication of this corrigendum, and are grateful to the Editor of *Molecular Medicine Reports* for allowing them the opportunity to publish this. They also wish to apologize to the readership of the Journal for any inconvenience caused.

## Figures and Tables

**Figure 3.**
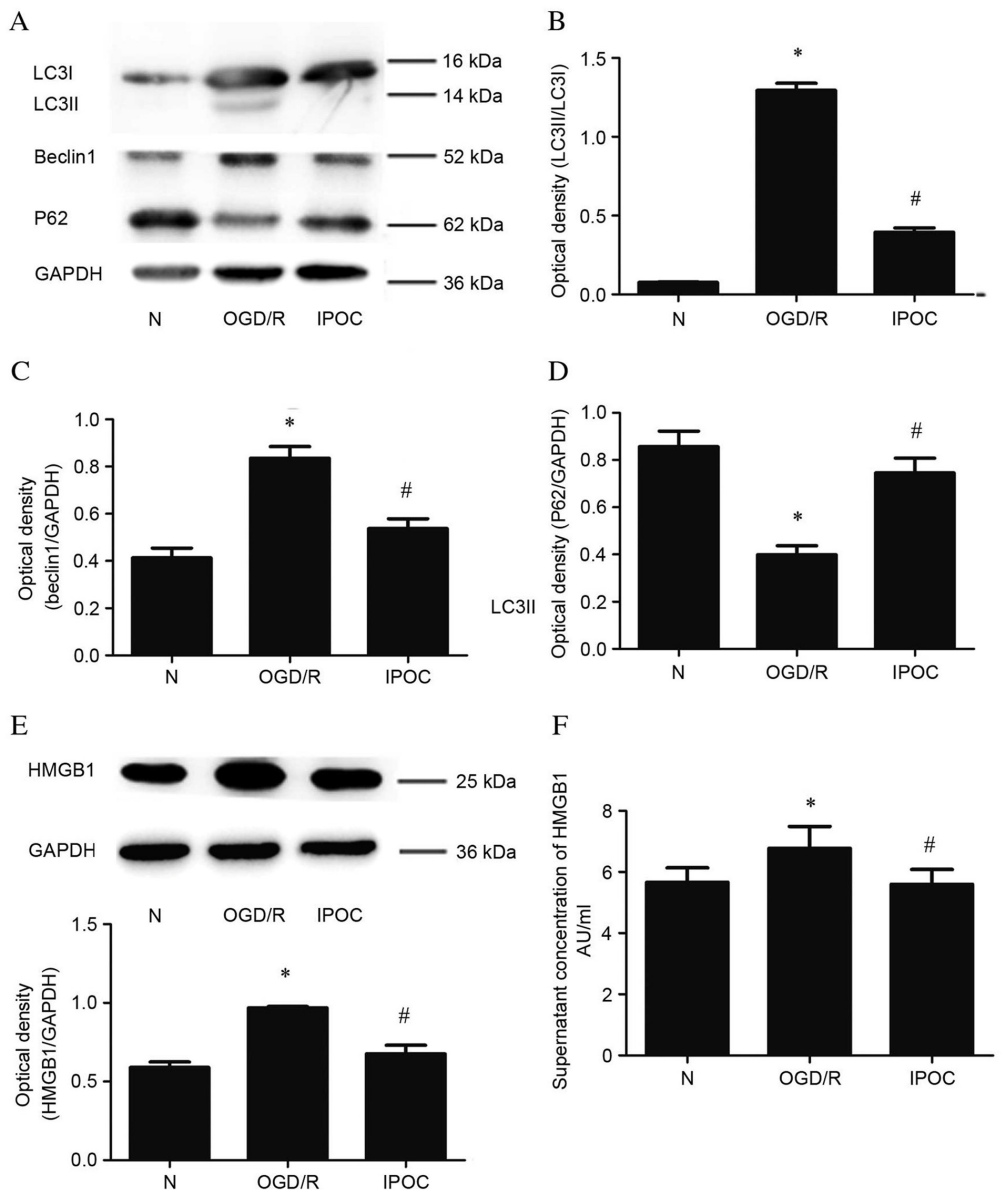
Analysis of protein expression levels of autophagy related proteins and HMGB1 following OGD/R (8 h OGD followed by 24 h reperfusion) and IPOC (OGD/R plus 3 cycles of IPOC). (A) Autophagy related proteins were detected by western blot; (B) quantitative analysis of LC3II protein relative to LC3I; (C) quantitative analysis of Beclin1 protein relative to GAPDH; (D) quantitative analysis of P62 protein relative to GAPDH; (E) cytoplasmic HMGB1 detected by western blot and quantitated relative to GAPDH; (F) quantitative analysis of cell supernatant HMGB1 protein detected by enzyme-linked immunosorbent assay. Data are presented as the mean ± standard deviation of 3 samples per group. *P<0.05 vs. normal control group, ^#^P<0.05 vs. OGD/R group. LC3, microtubule associated-protein 1A/1B-light chain 3; P62, sequestome 1; GAPDH, glyceraldehyde 3-phosphate dehydrogenase; HMGB1, high mobility group box 1; N, normal control group; OGD/R, oxygen and glucose deprivation and reperfusion; IPOC, ischemic post-conditioning.

**Figure 4.**
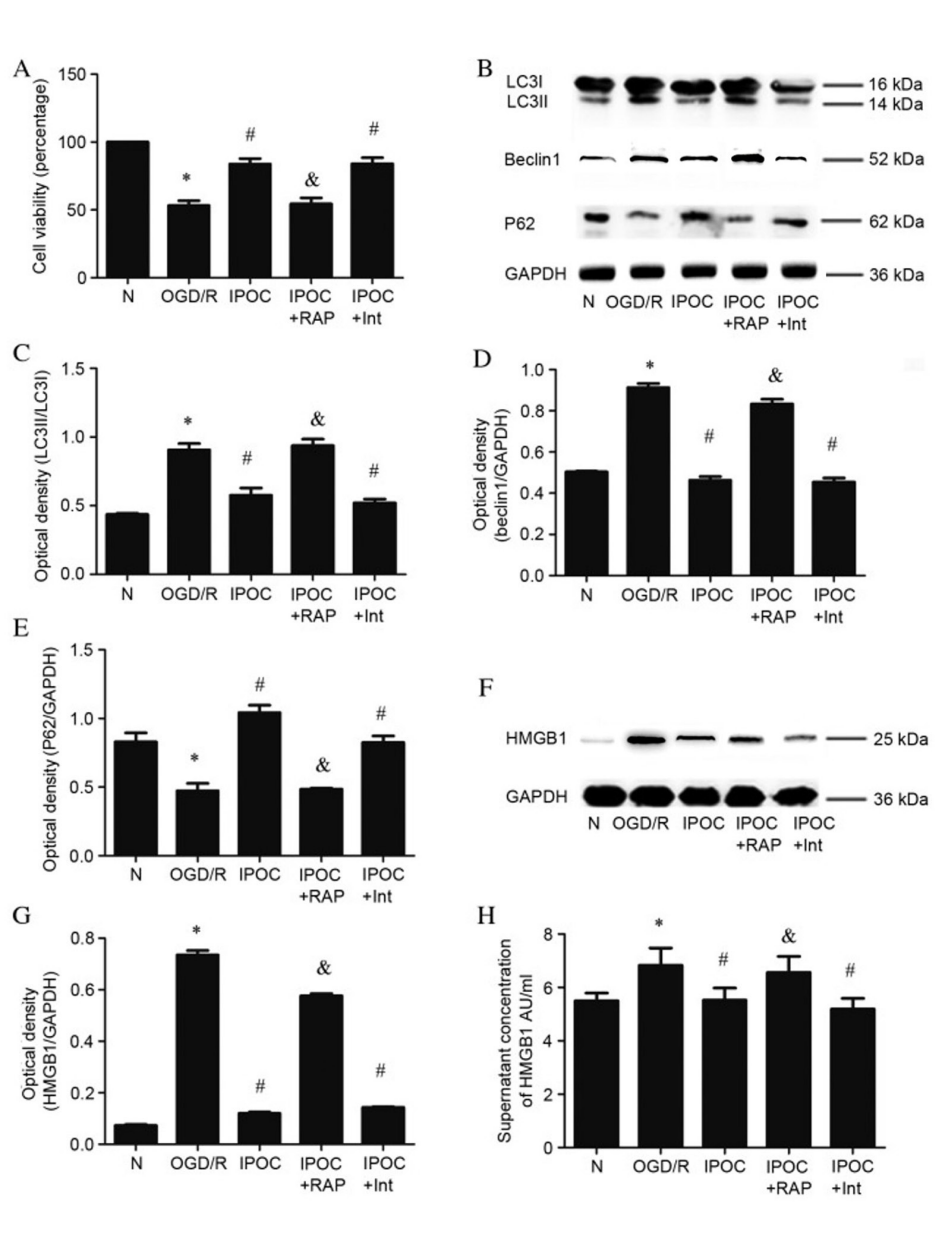
The effect of the autophagy activator, RAP, on IPOC (OGD/R plus 3 cycles of IPOC). (A) Cell viability evaluated using a CCK-8 assay; (B) expression levels of autophagy related proteins were detected by western blot; (C) quantitative analysis of LC3II protein relative to LC3I; (D) quantitative analysis of Beclin1 protein relative to GAPDH; (E) quantitative analysis of P62 protein relative to GAPDH; (F) cytoplasmic HMGB1 detected by western blot; (G) quantitative analysis of cytoplasmic HMGB1 protein relative to GAPDH; (H) quantitative analysis of cell supernatant HMGB1 protein detected by enzyme-linked immunosorbent assay. Data are presented as the mean ± standard deviation of 3 samples per group. *P<0.05 vs. normal control group, ^#^P<0.05 vs. OGD/R group, ^&^P<0.05 vs. IPOC group. N, normal control group; OGD/R (8 h OGD followed by 24 h reperfusion), oxygen and glucose deprivation and reperfusion; IPOC, ischemic post-conditioning; RAP, rapamycin; Int, Intralipid vehicle control; LC3, microtubule associated-protein 1A/1B-light chain 3; P62, sequestome 1; GAPDH, glyceraldehyde 3-phosphate dehydrogenase; HMGB1, high mobility group box 1.

**Figure 5.**
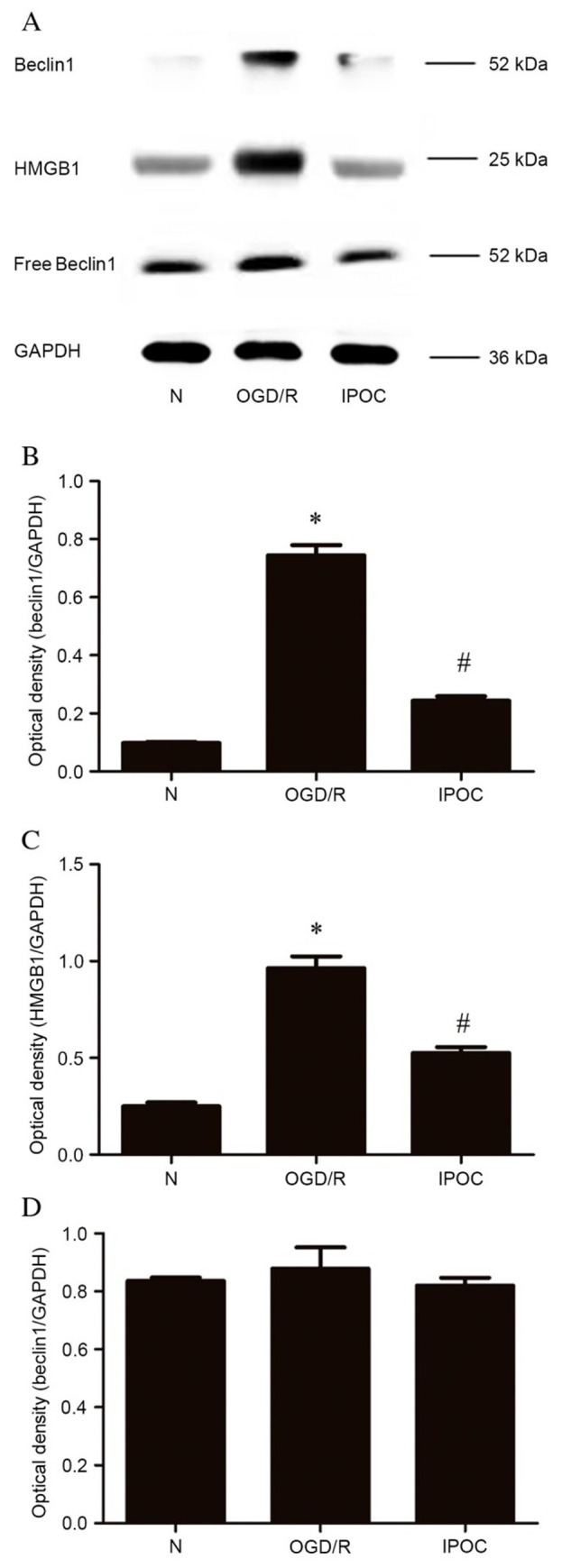
HMGB1 and Beclin1 co-immunoprecipitation. (A) Beclin1 and HMGB1 protein detected by western blot; (B) quantitative analysis of HMGB-bound Beclin1 relative to GAPDH; (C) quantitative analysis of HMGB1 protein relative to GAPDH; (D) quantitative analysis of free Beclin1 relative to GAPDH. Data are presented as the mean ± standard deviation of 3 samples per group. *P<0.05 vs. normal control group, ^#^P<0.05 vs. OGD/R group. HMGB1, high mobility group box 1; GAPDH, glyceraldehyde 3-phosphate dehydrogenase; N, normal control group; OGD/R (8 h OGD followed by 24 h reperfusion), oxygen and glucose deprivation and reperfusion; IPOC (OGD/R plus 3 cycles of IPOC), ischemic post-conditioning.

